# Medical deserts in Finland: measuring the accessibility and availability of primary health care services

**DOI:** 10.1186/s12913-025-12409-1

**Published:** 2025-02-19

**Authors:** Visa Väisänen, Markku Satokangas, Moona Huhtakangas, Harri Antikainen, Timo Sinervo

**Affiliations:** 1 Welfare State Research and Reform Unit, Health and Social Service System Research Team, Finnish Institute for Health and Welfare, Mannerheimintie 166, Helsinki, 00300 Finland; 2 Faculty of Social Sciences and Business Studies, Department of Health and Social Management, University of Eastern Finland, Yliopistonranta 8 E, Kuopio, 70210 Finland; 3 Faculty of Medicine, Department of General Practice and Primary Health Care, University of Helsinki, PO Box 20, Helsinki, 00014 Finland; 4 Faculty of Science, The Geography Research Unit, University of Oulu, Pentti Kaiteran Katu 1, Oulu, 90570 Finland

**Keywords:** Primary healthcare, Accessibility, Availability, Medical desert, Regional inequalities, Telehealth, Index

## Abstract

**Background:**

Well-functioning primary health care (PHC) systems are needed to meet the challenges of aging populations and increasing care needs. However, “medical deserts”, areas with poor accessibility and availability of PHC services, remain a significant issue throughout Europe, contributing to regional inequalities. Identifying the location of these areas is crucial for effective policymaking and for improving health outcomes. Our aim was to locate underserved areas in Finland by developing a medical desert index. In addition, we examined the impact of telehealth, care needs, and multiple funding sources on the index and analyzed its association with key quality indicators.

**Methods:**

The index was calculated using routinely collected municipality-level PHC consultation data from 2022 adjusted for population care needs (availability) and the average travel time to the nearest PHC center (accessibility). Telehealth and occupational healthcare consultations were included separately. Standardized index values were mapped and categorized using descriptive analysis, and compared with indicators of healthcare utilization, care accessibility and availability, care satisfaction, and continuity of care using correlation analysis.

**Results:**

The index displayed clear patterns of medical deserts, primarily in the rural areas of northern and eastern Finland. Approximately 13% of the Finnish population resided in medical deserts, defined as a standard score of -0.5 or lower. The inclusion of telehealth consultations appeared to improve the index values especially in some rural areas. Better accessibility and availability of PHC services, as indicated through the index, was significantly correlated with lower proportion of acute care consultations, fewer hospital care days, and lower continuity of care among clients aged 65 years and older.

**Conclusions:**

We were able to identify medical deserts in Finland utilizing novel methodology distinct from previous indicators, and thus providing important considerations for future research on regional inequalities in accessibility and availability of PHC services. Our findings demonstrated the potential of telehealth services in mitigating medical deserts, though its appropriateness for some population groups and care needs remains unclear. We call for health policy addressing PHC service provision especially in rural areas.

## Background

Healthcare systems across the world are facing challenges caused by aging populations [1], rising unmet care needs [2], and persistent unequal access to care [3]. Well-functioning primary health care (PHC) systems play a crucial role in alleviating and responding to these issues due to their role as the first point of contact for accessing healthcare [4, 5]. The essential components of modern PHC service delivery consist of continuous and coordinated care, which is both comprehensive and people and community-oriented [4]. This means that the care, encompassing preventive, curative, and rehabilitative services, is designed as the initial point of contact and is provided in close proximity to the local population. Although the importance of equal accessibility and availability of PHC services has been emphasized [6], this does not rule out systematic inequalities. Indeed, significant geographical variation in resourcing of PHC is a common finding [7, 8].

The accessibility and availability of PHC services can be defined in multiple ways but emerge as important prerequisites for better health outcomes. Penchansky and Thomas [9] define accessibility as the ability of potential service users to access services, including geographical and service-related factors such as travel and waiting times. Availability on the other hand refers to the adequate volume and supply of care resources, for instance the number of physicians in a specific geographical area [9, 10]. Better access to PHC has been associated with multiple key health outcomes, such as improved equity and population health, in addition to other important characteristics of care delivery, for example continuity and comprehensiveness of care [11]. Similarly, populations in rural and urban areas with poor access to care or high social deprivation have been found to have excess mortality [12]. It is also possible that inadequate accessibility and availability of PHC may further exacerbate the existing inequalities observed in selected health outcomes in rural areas [13]. Interestingly, only a small part of the regional variation in healthcare utilization can be explained by care needs and socioeconomic factors, potentially highlighting the significance of supply side factors [14, 15]. Access to healthcare can be viewed as resulting from the interaction between the sociodemographic characteristics of the population and the characteristics of the local services (e.g., location and quantity) [16]. To develop policy responses aimed at improving both the accessibility and availability of PHC services, it is first necessary to identify the locations of underserved areas.

Compared to the concept of access to care, medical desert refers to an area with both poor accessibility and poor availability of care [17]. While no formally accepted definitions exist, the term has been used mainly in relation to long waiting times and travel distances in addition to the low availability of healthcare workforce [17]. In a consensus-building exercise by Brînzac and colleagues [18], the following dimensions of medical deserts were established: insufficient human resources in health or facilities, long waiting times, high costs of services, and sociocultural barriers. A recent taxonomy developed for medical deserts highlighted the role of the economic resources of the population, among other factors [19]. In the literature, medical deserts are most often characterized by population-based attributes, such as population density or population size in relation to the number of health workforce, with significant variations in how the area is specified [20]. Determinants of medical desertification have also been explored, with health workforce planning and individual professionals’ preferences, in addition to organization and service system characteristics emerging as significant factors [21].

Efforts to locate medical deserts have focused on combining different measures of care supply, care demands, and travel times [22, 23]. With these relatively simple variables aggregated to a geographical area, information on potential medical deserts can be compiled. Consequently, medical deserts analyzed can emerge through an inadequate number of care professionals, or if the mean travel time to the nearest service provider is excessively high, or as a combination of these two. Importantly, the thresholds used and their definitions are based on comparisons between areas and may often be arbitrary. Recent work has aimed to incorporate population care needs and other service providers into the classification models [24]. These analyses have resulted in identification of medical deserts. In 2017, 18% of the French population lived in low-density priority areas (medical deserts) [17], and nearly 13% of the German population in 2010 resided in districts with the poorest health care accessibility, the majority of which were rural [25]. However, research from Ireland suggests that areas struggling with access to services (housing approximately 16% of the population) can also be located in more urban, often deprived, growth areas [26, 27].

One significant development during and after COVID-19 has been the rapid rise of digital health services [28]. From the perspective of PHC, the main interest lies in telehealth, where the care professional and the client communicate through remote means, as opposed to face-to-face consultations [29, 30]. In the context of medical deserts, functioning telehealth can improve the accessibility and availability of care and decrease regional inequalities, if residents can receive care anywhere and at any time. However, telehealth can also increase inequalities and complicate both the access to and provision of care, especially for individuals with poor technological skills [31–33]. As telehealth is being further integrated into healthcare systems, more evidence is needed on the long-term consequences for PHC and how telehealth can best complement and enhance the accessibility and availability of care, especially among marginalized populations and in rural areas.

### Finnish context

Finland and its mainly tax-funded health system are unique in their characteristics. The Finnish population is the third oldest in Europe and is increasingly aging [34]. Public primary health care provides universal coverage and is organized in healthcare centers, where the initial point of contact is often a registered nurse with a relatively wide scope of practice, including moderate drug prescribing rights. More than half of the care consultations in non-urgent public outpatient care centers are conducted by nurses [35]. General practitioners (GPs) act as gatekeepers to specialist care. The system involves relatively well-developed care integration between healthcare and social services [36, 37].

PHC services are also provided via alternative channels such as occupational health care (OHC), which offers medical primary care services (including GP consultations and specialist outpatient care) for 90% of employees [38]. While the services are funded mainly by the employer, they are partly covered by a national reimbursement scheme. Moreover, accessibility to GP consultations is better in OHC than in public PHC – i.e., OHC provides access to GPs with practically no waiting times or user fees, whereas public PHC has long queues and out-of-pocket payments [39]. This has been criticized for translating into wider inequalities between the population entitled to OHC and those who can use only public PHC. In addition, the use of private services, including voluntary health insurance (VHI), further fragments the care system based on individuals’ ability to pay.

Finland is one of the leading countries at the level of digitalization in the EU [40]. As nearly 33% (7.8 million) of all PHC outpatient consultations in 2023 (23.6 million) were conducted remotely [41], there is an opportunity to examine the potential of telehealth in PHC. While a majority of the remote consultations were conducted by nurses, GPs had over 2 million remote consultations in 2023. These numbers translate to approximately 3.3 remote consultations per PHC client during 2023, which might be a subject of regional and socioeconomic variation [42]. Geographically, Finland is a large country with a low population density of 18 per km^2^ compared with the EU average of 109 [43]. In Finland, 90% of the population lived within ten kilometers of a healthcare center in 2016 and PHC services were available within 20 min by car for 96% of the population [44, 45]. However, significant regional differences exist, with the accessibility being poorest in northern and eastern Finland [44]. Importantly, no further national analyses incorporating healthcare utilization have been conducted, underlining the need for more research. In addition, the population characteristics have significant regional variation, with the central and northern regions experiencing higher levels of morbidity [35]. Finland’s PHC also consistently ranks poorly in unmet care needs, indicating notable gaps between care needs and the accessibility or availability of care services [46]. The potential inequalities related to the accessibility and availability of primary health care might lead to the accumulation of adverse effects for underserved population groups.

As in many other European countries, the Finnish social and healthcare system has been under reform. In 1.1.2023, the new social and healthcare reform came into effect, which centralized social and health care in addition to rescue services to 22 new wellbeing services counties [46]. One of the aims of the reform was to decrease regional inequalities. However, the wellbeing service counties are facing high budgetary pressures, which is likely leading to the consolidation of healthcare center networks and the closing of hospitals and around-the-clock emergency departments. These developments, which are not entirely unique in Europe [2, 47], emphasize the need to gather baseline information on the potential regional inequalities in PHC services.

In line with previous research and policy initiatives mainly conducted in France [17], we have recognized the need to develop a medical desert index to examine the accessibility and availability of primary health care services in Finland. Such index, based on routinely collected healthcare register data, may have future potential in examining for example how the changes made during the pandemic (especially the wide-scale adoption of telehealth) and the worsening care workforce availability affect the end users of healthcare services and, more specifically, the accessibility and availability of PHC services. The index should also be assessed in relation to established quality indicators connected with deprivation of PHC services, such as the use of emergency services and perceived care satisfaction. Lastly, mapping potential medical deserts in Finland can help develop regionally specific measures to combat the determinants behind the gaps in PHC access and availability and ultimately help improve health outcomes.

Consequently, the following research questions were formulated:

1. What does the developed medical desert index reveal about the gaps in geographic distribution of PHC access and availability in Finland?
2. How do telehealth, care needs, and multiple funding sources affect the index?
3. How is the medical desert index associated with key health system and quality indicators?

## Methods

### Design

A cross-sectional study design with administrative care register data was utilized.

### Data

We used aggregated primary health care and specialized care consultations data per municipality in 2022 from the Finnish register of Primary Health Care visits and the Care Register for Health Care. The data were retrieved from the Statistics and Indicator Bank maintained by the Finnish Institute for Health and Welfare (sotkanet.fi). We included data on the physical and remote consultations of both physicians and nurses, with remote consultations consisting of both real-time (phone, video) and non-real-time contacts (mainly online chat).

The Finnish need-adjustment funding formula (the coefficients for health and social care needs) was used for care needs adjustment. This index combines information on individuals’ sociodemographic characteristics (such as age, disability pension, employment and socioeconomic status) and diagnoses with the average costs of treatment for these diagnoses [48, 49]. The obtained estimates of the social and healthcare public service costs of each resident are then aggregated to the level of the municipality, with a value of 1.0 acting as the average costs nationally.

Road network data from Esri Finland and 1 km × 1 km population grid cell data from Statistics Finland were used to determine travel times and travel distances to public healthcare centers in each municipality. The travel time and the corresponding travel distance were calculated as a population-weighted mean of the fastest driving routes between a population grid cell and the closest public healthcare center per municipality. Healthcare center data was from year 2018 with population characteristics from year 2022.

### Medical desert index

We calculated a medical desert index value separately for each municipality in mainland Finland. Medical deserts have been previously measured with an indicator comprising availability, accessibility, and demand for care [23]. More specifically, the availability of care has been measured via the number of GPs in each geographical area, accessibility with the mean travel time to the nearest GP (minutes with car) and care demands with the amount of population in each geographical area [22].

No explicit data on the number of physicians or nurses working per area or healthcare center are available for Finland. As such, to measure the availability of care, we chose care needs adjusted PHC use as a proxy for care supply, utilizing physician and nurse consultations from both PHC and OHC (Table 1). To account for population sizes, consultations per 1000 people were calculated. OHC consultations were included, as their role in primary outpatient care is significant among the employed [39]. Nurse consultations were accounted for half to consider the resource use of healthcare centers: In Finland, the wage of physicians is, on average, approximately 2.5 times that of registered nurses [50]. In addition, although registered nurses play a significant role in providing first point of contact care in Finland, only some nurse-led consultations can be performed without any consultation required from a physician.

**Table 1:** Comparison of measures used between previous work by Barlet and colleagues (2012) [22] and the present study

	**Previous work (Barlet et al., 2012)** [22]	**Present study**
**Supply**	Number of GPs	PHC physician and nurse^a^ care consultations OHC physician and nurse^a^ care consultations
**Demands**	Population (age and sex standardized)	Population size Care needs index
**Distance**	Mean travel time to the nearest GP by car	Mean travel time to the nearest primary healthcare center by car^b^
**Other**		Telehealth consultations were included

*GP* General practitioner, *PHC* Primary health care, *OHC* Occupational health care

^a^Nurse consultations were accounted for 50%

^b^Healthcare center location data from year 2018, with population characteristics from 2022

Care needs adjustment was based on the Finnish health expenditure index (more specifically, the coefficients for healthcare needs) [48]. The latest index value based on the most complete data (year 2022) was used. The index values varied from 0.78 to 1.42, with a mean of 1.00 (SD: 0.10).

Accessibility of care was measured via the mean travel time by car (in minutes) to the nearest healthcare center (locations from 2018). The mean travel time per municipality was compared to the overall median of mean travel times across all municipalities (6.8 min), leading to a variable with higher values indicating lower travel times and thus better accessibility. As a valid travel time was needed for the calculation, two different travel time configurations were assigned for the telehealth consultations conducted remotely. The first was the municipality’s average travel time, which simply equalizes the telehealth consultations with the physical consultations. The second was an arbitrary travel time of five minutes, signifying some level of friction with using the services (such as queues or waiting times). However, this relatively low travel time means that the impact of telehealth consultations on the index is greater for municipalities with higher mean travel times. To examine the potential of telehealth services, index configurations with and without telehealth consultations were visually compared. In addition, the two travel time configurations for telehealth were compared, but the travel time of five minutes was utilized in the consequent analyses, with the aim of further exploring and highlighting the potential of telehealth.

The index was constructed on the basis of the work of Barlet and colleagues [22], with the addition of taking care needs, nurse, OHC, and telehealth consultations into account.

Formula for the index value of a specific municipality: $y=\frac{\frac{1}{c }\left(a{d}_{1}+b{d}_{2}\right)-\mu }{\sigma }$

where:

y = Medical desert index value.

a = Physical (physician and nurse) consultations per 1000 people, including OHC.

b = Telehealth (physician and nurse) consultations per 1000 people, including OHC.

c = Care needs index: a value of 0.80 means that care needs are 20% lower (compared to the mean value of 1.00).

d_1_ = Travel distance compared with the median travel distance: a value of 0.75 means that the mean travel time is 33% higher (compared to the median value).

d_2_ = Travel distance of telehealth consultations (travel time of the municipality or 5 min).

μ = Mean value of the medical desert index.

σ = Standard deviation of the medical desert index.

The formula yields a standard score (Z-score), scaled in relation to the mean value of all the municipalities, indicating the number of standard deviations from the average value. A higher index score signifies better accessibility and/or availability of primary health care and a lower value indicates worse accessibility and/or availability of primary health care, both in relation to the average municipality. The effects of different parts comprising the index are demonstrated in Table 2. Higher mean travel distance and lower amount of care consultations especially affect the index value, while care needs have a smaller effect. The index consisting solely of physical consultations was less volatile and had lower variation, while the index including telehealth visits was more responsive to changes in telehealth visits and its travel time configuration.

**Table 2:** Sensitivity analysis on the hypothetical effects of different factors on the overall standardized index value (both physical only and including telehealth). An empty cell indicates that the mean/median value was used

**Hypothetical scenarios**	**Consultations per 1000 population**	**Mean travel time in minutes**	**Care needs index**	**Index value: physical only** Mean: 0	**Index value: incl. telehealth** Mean: 0
**1. Average**	Physical: 1991 Telehealth: 1222	Median: 6.8	Mean: 100.6	**−0.10**	**−0.08**
**2. Rural**		**25 min**		**−1.79**	**−1.40**
**3. Urban**		**4 min**		**1.51**	**1.18**
**4. Telehealth time equal to mean travel time**		Telehealth also 6.8 min		**−0.10**	**−0.48**
**5. Low care supply**	Physical: **1000** Telehealth: **600**			**−1.52**	**−2.13**
**6. High care supply**	Physical: **3500** Telehealth: **2500**			**1.65**	**2.87**
**7. High care needs**			**130.0**	**−0.51**	**−0.74**

These hypothetical examples are not representative of actual municipalities. The default travel time configuration for telehealth visits was 5 min, except for row 4

The municipalities were categorized into five groups, based on their standard scores: poor (< −1.5), low (−1.5 to −0.5), average (−0.5 to 0.5), good (0.5 to 1.5), and excellent (> 1.5). A standard score of −0.5 or less was designated as a medical desert, with the municipality likely struggling with poor accessibility and poor availability of PHC services. The categories were compared using population characteristics, such as population and population density, and in order to capture socioeconomic differences, the measure of yearly disposable household income per consumption unit (OECD measure) was used as a proxy.

### Quality measures

To explore how the created medical desert index functions and whether it is associated with different quality measures, multiple municipality-level indicators were retrieved from the Statistics and Indicator Bank maintained by the Finnish Institute for Health and Welfare (sotkanet.fi). The indicators encompassed healthcare utilization, care accessibility and availability, care satisfaction, and continuity of care. The indicators were chosen on the basis of previous research linking them to the accessibility and availability of PHC services.

For healthcare utilization, emergency department visits per 1000 people and the proportion of acute care consultations out of all PHC consultations were used. By acute care consultations, we refer to walk-in care received without an appointment in the PHC setting. In addition, hospital care days of treatment per 1000 people and the proportion of clients returning to the ED within 48 h were utilized. Both avoidable hospitalizations [51, 52] and emergency department care utilization [53, 54] have been previously associated with barriers related to the accessibility of primary care services.

Waiting times for physician (3 months and 7 days) and nurse services (7 days) were used to characterize care accessibility and availability. The indicators concerned the proportion of clients with a waiting time above 3 months or 7 days in ambulatory care in primary health care after the initial assessment of treatment needs. These indicators were chosen because timely access to treatment has been characterized as one of the key facets of care accessibility [18].

Care satisfaction, a common outcome of better care accessibility and availability [55], was measured via two indicators of client satisfaction in healthcare centers. One concerned receiving services in a reasonable time (timeliness), and the other concerned the usefulness of the services.

Lastly, continuity of care is an interesting concept that is especially relevant for telehealth services. However, research on this subject is scarce [56]. It was assessed for physician services using the Bice-Boxerman Continuity of Care indices (COCI) [57], which are calculated separately for the whole population of a municipality, for older people aged over 65, and for multimorbid clients. In essence, the indices describe the proportion of visits the target population has made to the same professional with a range of 0–1 [58].

### Statistical analysis

To statistically examine the created medical desert index, the correlation of index values with physical consultations only, and including remote consultations (with 5-min travel time), and selected indicators were tested. As all the variables analyzed violated the assumption of normality as tested by the Shapiro test, a nonparametric hypothesis test was used. Kendall correlation coefficients (τ) were calculated with a beta value of 0.05. Data management and statistical analyses were conducted using R version 4.3.2 [59].

## Results

The created medical desert index and the descriptive statistics of the original care supply (adjusted for care needs) and travel times are presented in Table 3. There were more physical consultations (mean: 1991) than telehealth consultations (mean: 1222) in the municipalities. For public PHC, telehealth consultations were one third of the overall consultations, while in OHC, telehealth consultations were over half of the total consultations. The travel time to the nearest PHC health center was on average 7.3 min, ranging from 1.7 to 31.5 min. As the index values were standardized, the mean was 0, and the standard deviation was 1.

**Table 3:** Finnish medical desert index values at the municipality level (*n* = 293). Consultation numbers are adjusted for care needs, by dividing them by the values of the care needs index. Nurse consultations accounted for half

Year 2022	Mean	Median	SD	Min – Max
**Physical consultations**	1991	1930	512	859 – 3509
Public PHC	1668	1645	511	514 – 3448
OHC	323	326	164	30 – 748
**Telehealth consultations**	1222	1182	525	181 – 3335
Public PHC	859	785	517	35 – 3271
OHC	363	361	189	30 – 924
**Travel time (minutes)**	7.3	6.8	2.9	1.7 – 31.5
**Travel length (kilometers)**	7.0	6.3	4.1	1.1 – 40.5
**Index value (physical only)**	0.00	−0.06	1.00	−1.96 – 6.00
**Index value (incl. telehealth)**	0.00	−0.03	1.00	−2.43 – 3.55

Supply numbers are presented in consultations per 1000 people. Travel time and length are by car to the nearest public healthcare center. “Index value (incl. telehealth)” is calculated with a travel time of five (5) minutes for the telehealth consultations

*PHC* Primary health care, *OHC* Occupational health care

**Fig. 1:**
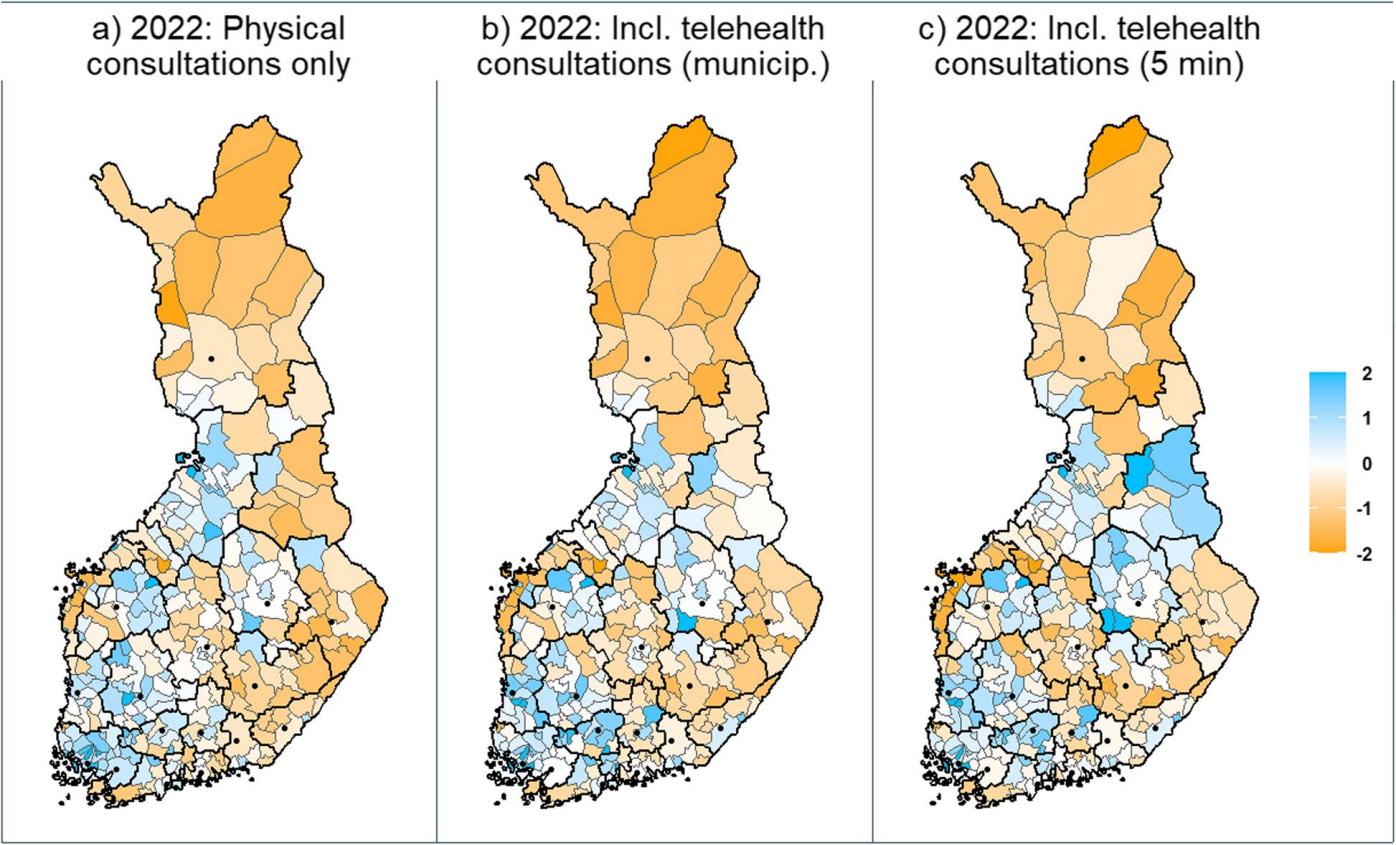
Medical desert index by municipality. Thicker lines depict wellbeing service county borders. The dots represent cities with population greater than 50 000. The orange color indicates lower index values, white average, and blue higher. Telehealth travel time calculated as municipalities’ average travel time in panel **b** and as 5 min in panel **c**

The values of the differently configured medical desert indices mapped across the Finnish municipalities can be seen in Fig. 1. On the basis of visual examination, areas with poor availability and/or accessibility of primary health care (medical deserts) appear to be concentrated in the eastern and northern Finland, and alongside the coastline. Coastal municipalities tend to have many islands, which significantly inflates the average travel times.

After including the telehealth care consultations with travel times equal to the municipality’s mean travel time, the availability and/or accessibility of care improved from poor to slightly below average in certain areas, especially in central and eastern Finland. If the travel time of telehealth consultations was set to five minutes, the situation improved further to very high accessibility and/or availability. Poor index values remained in some parts of the coastline and northern Finland. Importantly, while general trends and movement can be observed, the indices with varying configurations are not directly comparable, as the standardized values are calculated in relation to other municipalities for each panel.

**Fig. 2:**
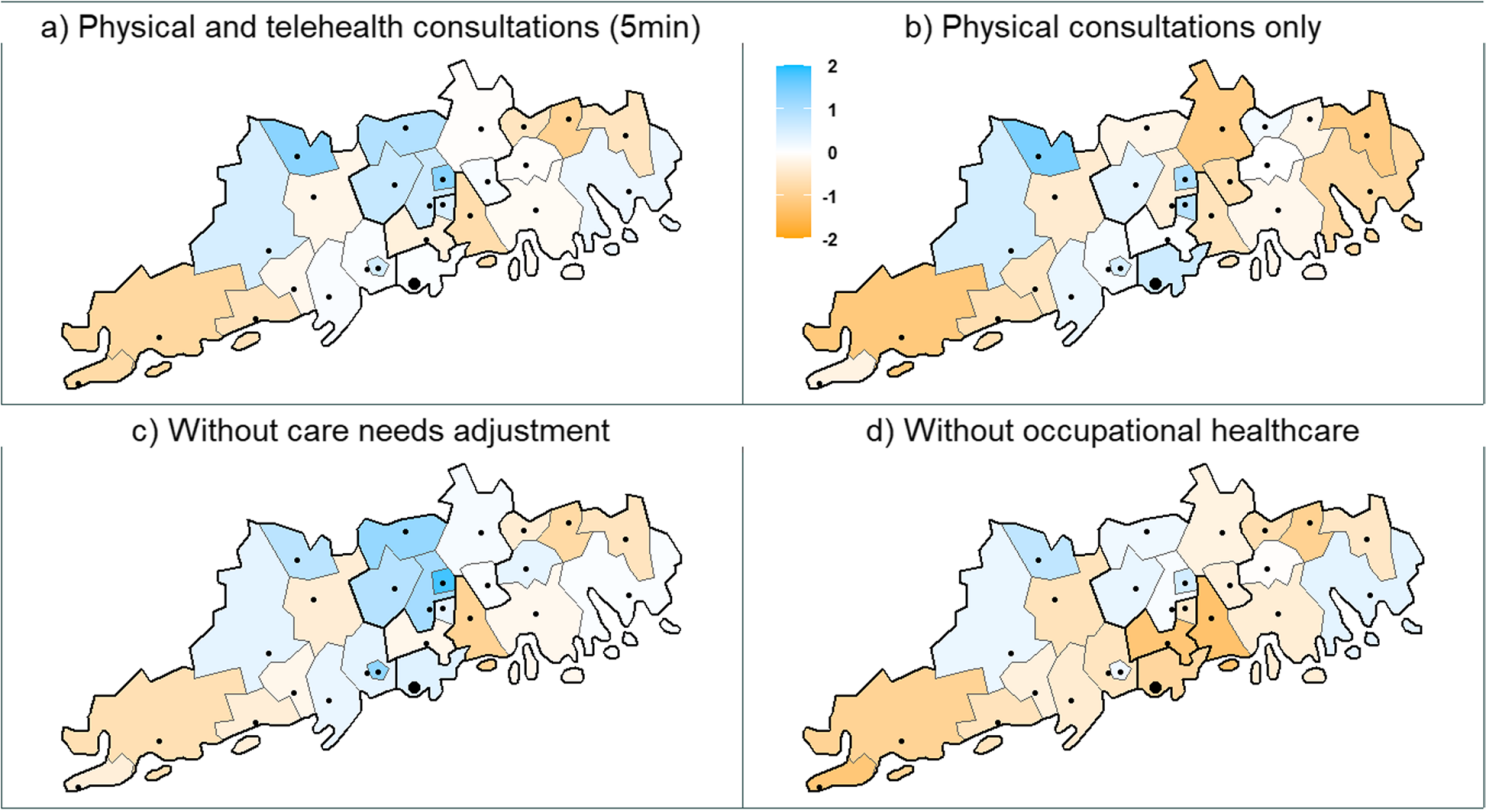
Medical desert index values by municipality in the southern Finnish region of Uusimaa. Thicker lines depict wellbeing service county borders. The dots represent cities, and the largest dot represents the capital city of Helsinki. The orange color indicates lower index values, white average, and blue higher. Panels **c** and **d** include telehealth consultations calculated with a travel time of 5 min

To highlight the effects of care needs adjustment and OHC, we concentrated on the Uusimaa region in southern Finland (Fig. 2). The Uusimaa region contains 26 municipalities, which in 2022 had approximately 1 733 000 residents, nearly one-third of the whole population. The region has a growing population, a considerable share of working-age residents, and a higher population density of 194 compared with 18 in the whole country.

In general, the region’s municipalities larger in size by area appear to have worse accessibility and/or availability of PHC, whereas the capital city and the neighboring municipalities have higher index values. After including telehealth consultations with a travel time of five minutes, the situation in some of the larger and more rural municipalities, with higher average travel times and lower population densities, improved significantly.

The effects of care needs adjustment and the inclusion of OHC consultations can be observed. While without care needs adjustment, the index values appear slightly better, without OHC the index in the region worsens significantly, signifying a high concentration of working-age population in the region. As can be seen from the last panel in Fig. 2, OHC is a significant PHC supplier, especially in larger cities.

Next, the municipalities were grouped according to the index values, which included the telehealth consultations with a travel time of five minutes (Table 4). Approximately 13% of the Finnish population (741 360 residents) lived in medical deserts, defined as areas with poor and low index values (Z-score lower than −0.5). Over a percent of the population lived in the municipalities categorized as the poorest in terms of care availability and/or accessibility. Municipalities with worse index values had significantly lower average population sizes and considerably lower population densities, and in addition, the mean disposable income in these areas was the lowest.

**Table 4:** Population characteristics at different levels of primary health care accessibility and availability as measured by the medical desert index (*n* = 293). The index including telehealth consultations with a 5-min travel time was used

Index value	Municipalities	Total population	Population	Population density	Mean income
	*n* (%)	*n* (%)	Mean (SD)	Mean (SD)	€ (SD)
**Poor** Under −1.5	17 (5.8%)	76 217 (1.4%)	4 483 (4 554)	6.6 (6.0)	27 803 (1 839)
**Low** −1.5 to −0.5	77 (26.3%)	665 143 (12.0%)	8 638 (12 270)	10.2 (12.7)	28 113 (2 452)
**Average** −0.5 to 0.5	116 (39.6%)	2 895 878 (52.3%)	24 964 (72 450)	66.0 (312.7)	28 742 (2 940)
**Good** 0.5 to 1.5	62 (21.2%)	1 653 568 (29.9%)	26 670 (49 287)	137.1 (323.4)	29 244 (4 953)
**Excellent** Over 1.5	21 (7.2%)	242 805 (4.4%)	11 562 (10 203)	55.5 (72.4)	28 426 (3 152)

Population density measured as population per km^2^. Income measured as the yearly disposable household income per consumption unit (OECD)

Approximately one-third of the population lived in municipalities with good or excellent care accessibility and/or availability. The municipalities with above average index values had much larger population sizes and higher population densities, in addition to higher mean disposable incomes.

Finally, we analyzed the associations between the created medical desert index and various municipality-level indicators of secondary care utilization, waiting times, patient satisfaction, and continuity of care (Table 5). While most of the indicators were not correlated with the index values, the proportion of acute care consultations among all PHC consultations, hospital care days of treatment per 1000 people and continuity of care for clients aged 65 years or over showed statistically significant, albeit weak, correlations.

**Table 5:** Associations between the Finnish medical desert index and select indicators of care utilization, accessibility and availability, satisfaction, and continuity of care

	**Medical desert index** Physical consultations	**Medical desert index** Also incl. telehealth^*^
	Kendall rank correlation coefficient, τ (*p*-value)	
**Care utilization** (*n* = 293)		
Emergency department consultations per 1000	−0.041 (0.293)	0.000 (0.997)
Proportion of acute care consultations out of all PHC consultations	**−0.138 (< 0.001)**	**−0.126 (0.001)**
Hospital care days of treatment per 1000	**−0.150 (< 0.001)**	**−0.115 (0.003)**
Clients returning to ED within 48 h (%)	−0.012 (0.758)	−0.056 (0.155)
**Care accessibility and availability** (*n* = 256–284)		
PHC over 3 months wait for physician^a^	−0.047 (0.357)	−0.048 (0.341)
PHC over 7 days wait for physician^b^	0.075 (0.073)	0.068 (0.106)
PHC over 7 days wait for nurse^c^	−0.001 (0.974)	0.023 (0.569)
**Care satisfaction** (*n* = 168)		
Healthcare center reception: Timeliness	−0.005 (0.921)	−0.001 (0.993)
Healthcare center reception: Usefulness	0.025 (0.655)	0.017 (0.754)
**Continuity of Care** (*n* = 270–286)		
Physician: All clients^d^	0.022 (0.578)	0.063 (0.117)
Physician: 65 + year old clients^e^	**−0.111 (0.007)**	−0.078 (0.060)
Physician: Multimorbid clients^d^	0.029 (0.473)	0.064 (0.109)

*PHC* Primary health care, *ED* Emergency department

n-values differ, ^a^ = 256, ^b^ = 257, ^c^ = 284, ^d^ = 286, ^e^ = 270

^*^Travel time of telehealth consultations was defined as 5 min

Better accessibility and availability of primary health care, as measured by the index, correlated negatively with the proportion of acute care consultations out of all PHC consultations (τ = −0.14 for physical consultations and τ = −0.13 including telehealth). In addition, lower hospital care days per 1000 people were associated with higher index values (τ = −0.15 for physical consultations and τ = −0.11 including telehealth). The index was also correlated with lower continuity of care (physician consultations) for older people (τ = −0.11 for physical consultations).

## Discussion

In this study, we present a Finnish medical desert index with the aim of determining the location of areas with low accessibility and/or availability of primary health care services, in relation to other municipalities. We utilized routinely collected healthcare data and incorporated information on travel times, population care needs, telehealth services, and OHC services. Approximately 13% of the population resided in medical deserts or areas defined as having low or poor accessibility and/or availability of PHC services. Medical deserts appear to be located especially in eastern and northern Finland and alongside coastal areas. The inclusion of telehealth services in the index seemed to improve the accessibility and/or availability of PHC, especially in some underserved areas that had greater utilization of telehealth. The index functioned in a consistent manner, showing relatively clear geographical trends and concentration of primary services around urban centers. The index values correlated negatively with the proportion of acute care consultations, hospital care days and continuity of care among clients aged 65 years or over, whereas the associations with other measures of care utilization, accessibility and availability, and satisfaction remained statistically insignificant.

The developed index is the first of its kind in Finland and showcases a novel attempt to use routinely collected healthcare data for mapping potential gaps in the accessibility and availability of local PHC services. The present study provides national and local policymakers with a tool that indicates areas struggling with such gaps and may help these authorities identify the most relevant interventions for addressing them. Such interventions could include, e.g., addressing health workforce issues [20] or innovative solutions for organizing service delivery [60, 61]. Eventually, addressing PHC accessibility and availability in the identified areas could also translate to better health outcomes and decrease regional inequalities.

In principle, the relative index identifies municipalities where the average travel time to the nearest public healthcare center is above average and/or there is a below average throughput of primary care services when accounting for population care needs. While unlikely, it is possible that the poor performance in the index of some municipalities could stem from the utilization of novel models of care, which results in relatively low numbers of care consultations and/or high average travel times. One example could be mobile care units [62], which the new wellbeing services counties have been experimenting with. More specific aspects of the local PHC systems, such as models of care, were not taken further into account due to data constraints. Therefore, the index values of the municipalities could have determinants outside the ones included in the formula. However, the index shows consistent geographic trends and offers baseline information on the potential location of Finnish medical deserts before the large reform, which can enable further analysis of care accessibility and availability or reform outcomes. In addition, it makes subsequent longitudinal analysis and tracking of trends possible.

The geographical trends displayed in the developed index are mostly in line with previous research on the catchment areas of Finnish PHC centers [44, 63], which is unsurprising, as the analyses utilize identical travel time data. However, no domestic research that incorporates care supply or healthcare consultations exists. The results are supported by the current views and findings of differences between Finnish urban and rural areas [64], which present northern and eastern Finland as areas struggling with poor accessibility and availability of care. Our findings indicate that approximately 13% of the population resides in areas with low or poor accessibility and/or availability of PHC, as determined by an ultimately arbitrary index value cutoff of −0.5 or less (Z-score). This is similar in magnitude compared to results from international research, with 18% of the population in France [17] and 13% in Germany [25] living in areas with poor accessibility or availability of care. Importantly, the various indicators differ significantly in how they are calculated, and most have care supply information, such as the number of GPs working in a specific area. As these data are not available in Finland, we used the number of care consultations per 1000 people as a proxy for care supply. As the Finnish PHC system has long suffered from availability issues [35], it can be assumed that the current care supply is extensively utilized. In addition, it can be argued that the use of consultation numbers could more accurately capture the supply of care if population care needs are sufficiently controlled for. The care supply provided by each GP could potentially vary significantly, depending on, for example, telehealth service use and population morbidity. In addition, by using consultation data, we were able to capture the care provided by nurses and via alternative care pathways (occupational health care). Furthermore, the use of this method enabled the incorporation of telehealth consultations with differently, albeit arbitrarily, assigned travel times. This is especially relevant if telehealth services are provided by distinct actors located geographically away from residents. However, sensitivity analyses comparing the different data sources and methodologies should be conducted, if or when both are available.

The present study investigated how telehealth services could respond to the issue of medical deserts. Higher telehealth use is directly reflected in the medical desert index through more care consultations resulting in greater availability of care, and the lower travel time of telehealth consultations leading to better accessibility of care. This is supported by previous research, which has shown the potential of telehealth services in improving both the accessibility and availability of care, in addition to other positive outcomes [29, 65, 66]. While our results highlight the possibility of telehealth improving both the accessibility and the availability of PHC services, for some clients, especially those of older age, the appropriateness of telehealth remains debatable given the high risk of digital exclusion [67, 68]. A recent systematic review reported mixed results concerning general practice telehealth use, with both younger working-age people and the very old being more likely to use services [69]. However, low digital competence has been found to partly mediate the association between poor access to healthcare and the utilization of services [31], indicating that the potential benefits of telehealth might not be equally distributed across different demographic groups. Additionally, the appropriateness of telehealth for treating certain, especially chronic, conditions remains unclear [70]. Lastly, while well-implemented telehealth services are likely to improve the accessibility and availability of primary care services, concerns have been raised about the potential negative effects on patient-centeredness and continuity of care [71, 72]. This is especially relevant if telehealth services are organized separately from PHC services in distinct digital care units, in which case efficient collaboration among different care professionals, including unhindered information transfer, becomes essential [71]. Consequent studies should strive to explore whether the rapidly growing telehealth use in primary health care is enhancing the accessibility and availability of care, in addition to improving health outcomes, equally across all population groups, or if the overall benefits accumulate among those more receptive to digital health services.

We were able to consider both population care needs and OHC consultations in the developed indices. First, the care needs of the population should be accounted for when analyzing medical deserts, as the number of care consultations (or GPs in other studies) should, at least in theory, be proportional to the population care needs. If care needs are not accounted for, the index values can appear lower or higher based on the population characteristics, as could be seen in our results. Some previous indicators have standardized the population sizes for age and sex [22] or used median income levels and mortality rates as proxies for socioeconomic level and care needs, respectively [24]. If available, existing formula funding schemes [73] or risk adjustment methods [74], which estimate healthcare use or costs at the individual level, should be utilized (aggregated to the area level). Second, the inclusion of OHC consultations offers a novel view into the role of alternative service channels in measuring the accessibility and availability of PHC services. Without OHC consultations, we found that the index values in the municipalities of Uusimaa region with a high proportion of working-age people appeared much worse, as the large amount of substitutive PHC use was not accounted for. The same could apply to VHI schemes or private services in other countries, which future medical desert analyses should consider. In addition, the equity-related aspects often related to alternative care pathways should be further analyzed [75, 76].

Despite providing a plausible picture of the gaps in PHC accessibility and availability, the developed medical desert index was associated with only some of the chosen PHC quality indicators. It is likely that, beyond the accessibility and/or availability of PHC, various other determinants contribute to the chosen indicators. For example, client satisfaction was measured from clients who have accessed care, and accessibility (waiting times, queues) is only one aspect of client satisfaction. In addition, the index did not include measures such as actual waiting times or perceived care quality, which may differ significantly from the calculated accessibility and/or availability of care. However, three indicators were statistically significantly, although weakly, correlated with the medical desert index. First, higher index values (i.e., better access and availability) were associated with a lower proportion of acute care consultations out of all PHC consultations. Acute care consultations refer to urgent walk-in care received in PHC without an appointment. Residents living in medical deserts might resort to the use of such services if the PHC center suffers from high waiting times for an appointment. This is corroborated by previous research, which has linked poor PHC access to increased use of urgent care clinics, largely motivated by convenience and the absence of better alternatives [77, 78]. Second, higher values of the medical desert index were correlated with fewer days of hospital treatment (per 1000 people). This result is in line with the calls to invest in PHC services to reduce strain on specialized hospital care, which tends to lead to worse health outcomes and higher costs. Accessible PHC services have been previously associated with lower avoidable hospitalization rates [51, 52], underlining the importance of identifying and addressing medical deserts. Third, the index was negatively associated with continuity of care among 65 + year old clients. This somewhat surprising finding highlights the potential conflict between continuity of care and increasing the accessibility of care, which can be achieved through, for example, the hiring of (temporary) physicians, task-shifting of responsibilities to other care professionals, or digital care services. These practices may inherently decrease continuity of care, as PHC services through alternative channels and additional professionals become available. While continuity of care is especially important for older clients, who generally tend to have greater care needs [79, 80], a delicate balance needs to be found between adequate patient-centeredness and continuity of care and sufficient accessibility and availability of care. Overall, the statistically significant correlations indicate that the developed medical desert index may capture some dimensions of PHC quality.

The results have implications for the recent Finnish reform and for future or ongoing healthcare reforms internationally. The national reform is currently in its third year, as the wellbeing service counties are facing heavy budgetary pressures, with many drafting plans for consolidating their PHC network, including closures of healthcare centers. If these changes to the service network concentrate on the already underserved areas, such as the medical deserts established in the present study, the accumulating effects could further exacerbate regional inequalities. If necessary, changes to the service network should focus on areas with relatively high care supply and/or short travel times. Importantly, as OHC is not provided by the wellbeing services counties, service changes likely disproportionately affect those not covered by the OHC scheme, namely older people. As one of the official goals of the reform was to reduce regional inequalities, monitoring the outcomes of the reform and the changes made to the PHC service network, especially from the point of view of medical deserts, is essential. For healthcare reforms in other countries, the developed medical desert index serves as an example of a relatively easy-to-calculate measure that can help locate areas with poor accessibility and availability of care services. As the austerity policies in the European Union have reduced access to healthcare, particularly among vulnerable groups [81], future reforms should strive to strengthen PHC services, especially in rural areas. As demonstrated in the present study, the chosen methodology for analyzing medical deserts can be tailored to match the data availability and the health system of the subject country. With the prevalence of small-scale reforms emphasizing task-shifting, multiprofessional models, and telehealth services, including additional care supply in the calculations becomes of greater relevance. This is especially important, as many of the approaches proposed to mitigate medical deserts focus on digital care services and the utilization of other care professionals [20, 21]. We call for further international research on the issue of medical deserts, as systematically mapping out areas facing poor accessibility and availability of PHC services can help develop measures to combat the determinants of medical deserts and importantly stimulate healthcare reforms with the aim of strengthening PHC services for all.

### Limitations

The developed medical desert index has several aspects that limit its interpretability. First, the index is relatively rudimentary, as it simplifies the complex phenomenon of PHC accessibility and availability into care consultations per resident adjusted for travel times and population care needs. For example, the index did not account for residents without access to cars or include specialized and urgent care. In addition, the used formula required assigning a valid travel time for telehealth consultations, and an arbitrary value of 5 min was used for all municipalities. While this allows for some waiting times and queues, it means that regional variation in the accessibility to telehealth services was not accounted for. However, centralized digital health clinics and the prominent role of nurses in the healthcare system should support timely access to telehealth services.

Next, the index was calculated using the care consultations over one year. This has the benefit of convenience and diminishes the effects of random variation in care utilization, especially in smaller municipalities. However, PHC care utilization is not constant throughout the year, which means that the severity of medical deserts could vary seasonally. In addition to patterns of flu-seasons [82], the use of health services can be affected by holiday seasons and tourism [83, 84], which can further aggravate the availability of PHC services in medical deserts. This could potentially bias the index values upward, as the consultations were calculated in proportion with the permanent residents of the municipality and did not include, for example, temporary vacationers or tourists.

Compared with previous medical desert indicators, the present index has a different definition of care supply. We had no data on the number of physicians or nurses working in a healthcare center or a municipality, and as such, we chose to use the number of consultations as a proxy for care supply. This may affect the comparability and interpretation of the index values. While this method has several strengths, for example, being able to distinguish between physical and telehealth consultations, it does not directly measure the health workforce situation, which is a major determinant of medical deserts [18, 20]. This can also affect the index values if care models or documentation practices of a municipality differ significantly, for instance, if average consultation lengths are longer or if the accessibility to telehealth services is poor. Future analyses would benefit from being able to differentiate between shorter and longer consultations.

The travel time was calculated as the mean travel time of the population by car, as opposed to more sophisticated floating catchment area techniques [85], which can account for service demands, capacity, and multiple transport modes. However, previous research has indicated that the accessibility of primary care measured by both car and public transport are similar in trend [44], suggesting that the present results could also be applicable to other modes of travel. In 2022, municipalities acted as the main care providers for their residents, and the utilization of choice (residents changing their PHC center), while possible since 2014, has remained relatively uncommon [35]. This indicates that a vast majority of the population received PHC services mainly in their municipality of residence. Consequently, the potential for edge effect (failing to account for behavior outside the study area), which is a commonly cited problem in geographical accessibility research [86, 87], may not be a major concern in the present study.

The inclusion of OHC consultations strengthened the index, especially when considering the significant role of the system in Finland. However, the travel time used was calculated for the nearest public healthcare center, which is not where OHC is provided. We assume that both public and private healthcare providers are located mostly in population centers, but in practice, travel time could differ for PHC and OHC consultations. Next, while standardizing the medical desert index facilitates the interpretation of the results, it also means that for different years, the variation in the index and thus the absolute threshold for the medical deserts changes, which complicates year-to-year comparisons. In addition, the results were standardized in relation to the other municipalities, which means that the index always produces better and worse areas. Finally, as is common with routinely collected register data, variations in documentation practices or care models used, along with the amount of missing data, can impact the results of a single municipality. As such, the results concerning a single municipality should be interpreted with care.

## Conclusions

We present a novel attempt at mapping out the medical deserts in Finland, which refer to areas with poor accessibility and availability of PHC services. In contrast with previous international studies, we relied on routinely collected consultation data, which allowed us to incorporate telehealth services, population care needs, and nurse and occupational healthcare consultations. The developed index revealed clear trends in medical deserts, which were located primarily in the rural regions of northern and eastern Finland but also alongside the coastline. Approximately 13% of the population lived in medical deserts. Unsurprisingly, the index values were highest in and around larger cities and urban centers. The inclusion of telehealth services appeared to improve the situation in some, especially rural, areas. The index values were associated with urgent care and hospital service utilization, suggesting that the index also functions as a proxy for some dimensions of PHC quality. In addition to methodological considerations, our results support policymakers with information on the accessibility and availability of PHC services and can help develop regionally specific tools to mitigate medical deserts. In the aftermath of the recent Finnish social and healthcare reform, extensive changes to the PHC service network have been proposed. The present study provides a baseline for monitoring these changes and enables analysis of their determinants and consequences for the accessibility and availability of PHC services, which form the cornerstone of functioning healthcare systems.

## Data Availability

The datasets used and/or analyzed during the current study are available from the corresponding author on reasonable request.

## Abbreviations

COCI: Continuity of care index
ED: Emergency department
GP: General practitioner
OECD: Organization for Economic Co-operation and Development
OHC: Occupational health care
PHC: Primary health care
SD: Standard deviation
VHI: Voluntary health insurance
